# Protein Folding Interdiction Strategy for Therapeutic Drug Development in Viral Diseases: Ebola VP40 and Influenza A M1

**DOI:** 10.3390/ijms23073906

**Published:** 2022-03-31

**Authors:** Fernando Bergasa-Caceres, Herschel A. Rabitz

**Affiliations:** Department of Chemistry, Princeton University, Princeton, NJ 08544, USA; hrabitz@princeton.edu

**Keywords:** Ebola, influenza, folding, pathway, interdiction, therapeutic, drug

## Abstract

In a recent paper, we proposed the folding interdiction target region (FITR) strategy for therapeutic drug design in SARS-CoV-2. This paper expands the application of the FITR strategy by proposing therapeutic drug design approaches against Ebola virus disease and influenza A. We predict target regions for folding interdicting drugs on correspondingly relevant structural proteins of both pathogenic viruses: VP40 of Ebola, and matrix protein M1 of influenza A. Identification of the protein targets employs the sequential collapse model (SCM) for protein folding. It is explained that the model predicts natural peptide candidates in each case from which to start the search for therapeutic drugs. The paper also discusses how these predictions could be tested, as well as some challenges likely to be found when designing effective therapeutic drugs from the proposed peptide candidates. The FITR strategy opens a potential new avenue for the design of therapeutic drugs that promises to be effective against infectious diseases.

## 1. Introduction

Very significant progress has been made in recent years in the field of therapeutic drug design through the application of innovative experimental techniques, relying on a growing understanding of the molecular basis of disease and improved computational capabilities including, most recently, artificial intelligence [1,2]. Most current approaches to drug design are based on identifying particular functional targets on the folded structure of relevant proteins of pathogenic organisms [3]. Such targets include enzymatic active sites [4], receptor binding sites [5], and allosteric regulation regions [6].

Despite considerable successes, the goal of a comprehensive approach to drug design that can provide effective therapeutic molecules for most human infectious diseases remains elusive [7]. The reasons are manifold, some related to insufficient understanding of the molecular basis of many diseases [8], others to the difficulty of designing therapeutic molecules that effectively interfere with the functionality of fully folded proteins [9], or the presence of post-translational modifications of key proteins [10]. Additional difficulties are involved in delivering therapeutic molecules, particularly inside a cell [11]. Perhaps, most importantly, the continuous evolution of viruses and bacteria makes it difficult to design drugs and vaccines whose efficacy persists over time [12,13], which is a particular issue very relevant in the case of fast-mutating RNA viruses [14].

In the context of the SARS-CoV-2 pandemic, we recently proposed a therapeutic drug design strategy based on inhibiting the early folding process of the target pathogenic protein, rather than its fully folded form [15,16]. The strategy is based on the identification of specific regions on the protein primary sequence that are essential for the formation of the first contacts along the folding pathway. Then, specific molecules can be designed such that formation of the initial protein contacts is inhibited thereby preventing the protein from folding. Thus, the proposed approach is called the folding interdiction target region (FITR) strategy for drug design.

In order to predict the earliest folding events along the folding pathway of two SARS-CoV-2 proteins and provide for potential FTIRs for both, we employed the sequential collapse model (SCM) [17]. The SCM provides predictions of the relevant segments of the FITR on the basis of a relatively simple coarse-grained methodology [17]. The FITR proposal is conceptually similar to previous work exploring the possibility of employing relatively simple protein folding models to search for potential drug targets [18].

Besides the current ongoing SARS-CoV-2-related pandemic and its horrific human toll, there are a number of viral diseases with which humanity has coexisted for a long time that regularly cause considerable levels of sickness and death whenever a serious outbreak occurs. Two prominent examples of such diseases, both caused by RNA viruses such as SARS-CoV-2, are influenza [19] and Ebola [20]. Influenza infects many millions each year and causes up to ~650,000 deaths [21]. Moreover, influenza, due to continuous mutation and antigenic drift combined with important avian zoonotic reservoirs, has significant pandemic potential [22]. Ebola is one of the most lethal diseases that affects humans, with lethality rates of up to ≈90%. Underlying its high lethality, Ebola has a very efficient mechanism to evade the immune system by cloaking itself in fragments of the host’s own cell membranes [23], making it hard to specifically target. Moreover, Ebola also has a zoonotic reservoir in bats [23]. For these reasons, we have chosen influenza and Ebola as two strong examples to further demonstrate the potential applicability of the FITR strategy. The choice is not in any way meant to imply that there is any degree of commonality between the two diseases beyond what is generally understood [24].

**Figure 1:**
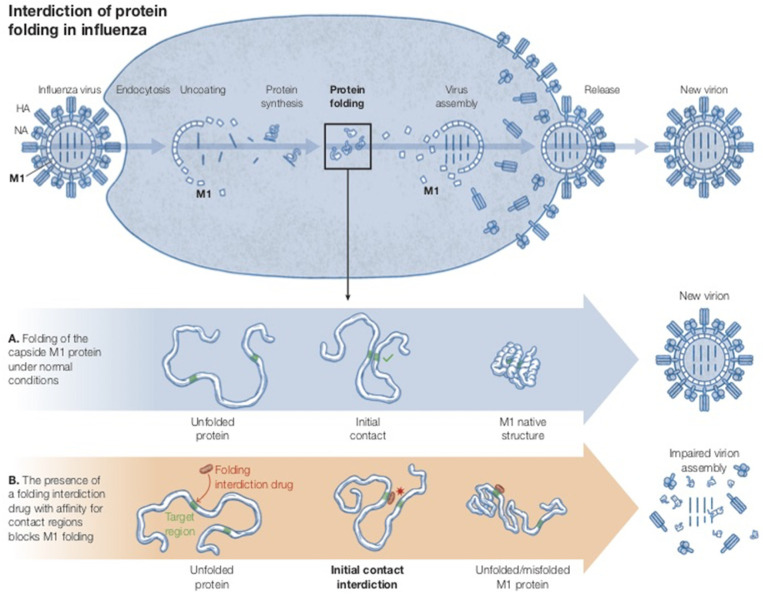
Illustration of the FITR strategy aimed to disrupt viral assembly by targeting critical structural proteins; the case of influenza protein A is depicted.

In this paper, we further demonstrate the potential applicability of the FITR strategy across these two diverse viral diseases by predicting the initial contact formation events along the folding pathway of two domains of proteins of important therapeutic significance: (a) the N-terminal domain of the VP40 protein of the Ebola virus (EBOV), and (b) the N-terminal domain of the M1 matrix protein of the influenza A virus. Both VP40 and M1 are critical to defining and maintaining the morphology of their respective viruses, and thus are very relevant to their functionality [25,26,27,28,29,30]. Moreover, both proteins are highly conserved and have been considered promising mutation-resistant therapeutic targets [31,32,33,34]. Thus, the predicted contacts are expected to constitute good FITRs for therapeutic drug design in both cases. Moreover, by targeting structural proteins involved in the definition of the overall virus morphology, we provide for simple proof-of-concept testing of the proposed therapeutic design strategy as the effects of the drugs should be noticeable in high-resolution microscopy imaging of the virus [35] and not just in more subtle activity measures. Besides predicting the FITRs for both proteins, we also further discuss the practical applicability of the findings to therapeutic drug design, as well as the steps that could be taken for its experimental testing. The FITR strategy as intended for the proteins studied here is generically depicted in Figure 1.

It is important to point out a main difference between the FITR strategy proposed here and the well understood drug design strategies that target the folded protein, usually its active/binding site: importantly, *the FITR strategy targets the unfolded protein aiming to pre-empt the folding process altogether*. Such an approach has the potential advantage of being amenable to simpler analysis than offered on interacting with the fully folded protein. However, the FITR intracellular constraint that the therapeutic (i.e., likely a peptide) drug must be delivered specifically close to the cell ribosomes presents a feasible yet challenging approach [36]. 

The FITR strategy remains experimentally untested, although efforts are underway to fill this gap. The purpose of this paper is further dissemination of the concept, such that more varied experimental tests might take place over a broader range of proteins belonging to viruses other than SARS-CoV-2. There might be considerable complexity associated with bringing the application of the FITR strategy to the end result of obtaining effective intracellular therapeutic drugs. Even if those challenges take substantial time and effort to overcome, the SCM-based FITR strategy could provide a powerful new tool to fight disease.

## 2. Results

### 2.1. The Physical Basis of Non-Local Early Contact Formation in the SCM

The physical basis of the SCM and its most up-to-date formulation have been recently explained in detail [17], and the associated calculational methodology is summarized in detail in the Section 3 of this paper. Here, a brief introduction to the main concepts is presented that is relevant to the issues investigated in the present paper. The SCM considers early non-local contacts on the basis of the entropy of formation of the resultant protein loops in the unfolded state, as well as the hydrophobic stabilization energy of the protein segments that define the contacts. The SCM has successfully predicted many of the observed features of protein folding pathways at low resolution [17]. Within the SCM, the folding of proteins with more than ≈100 amino acids is nucleated by the formation of a specific early non-local contact, called the primary contact, that defines the earliest folding phase. Primary contacts between two protein segments centered at residues i and j, separated by a distance along the protein sequence n_ij_, form at an optimal distance n_op_ such that n_ij_ ≥ n_op_ ≈ 65 amino acids, where the actual contact at n_ij_ is determined by the excluded volume-related entropic consequences of forming early protein loops [17].

**Figure 2:**
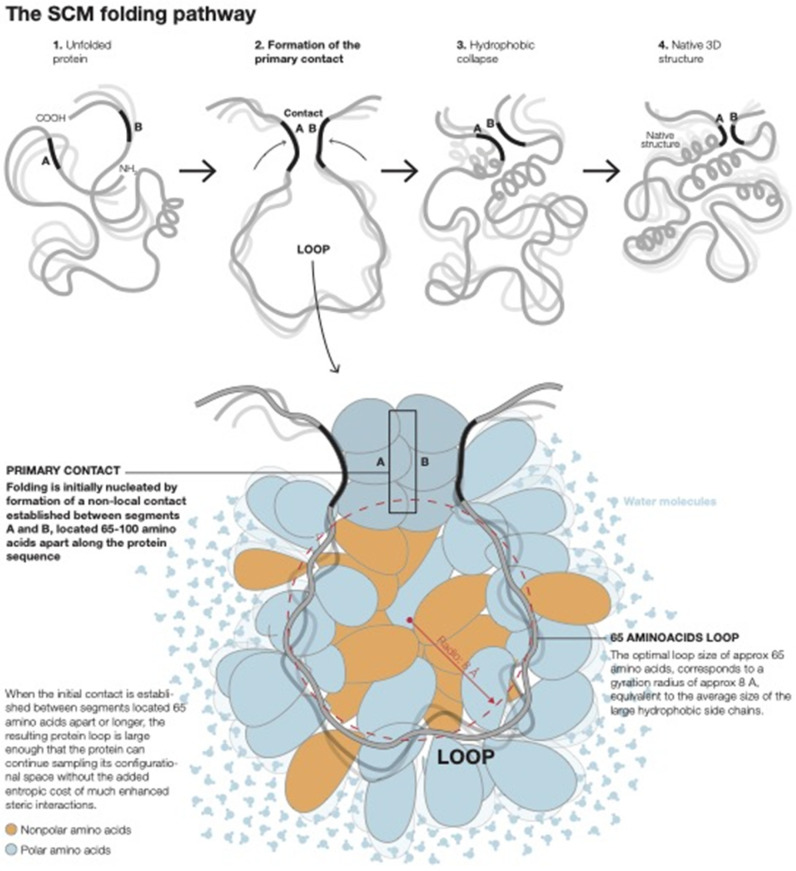
The physical basis of the SCM early non-local contact formation.

For a given protein sequence, there might be several viable primary contacts that nucleate parallel folding pathways [17]. As at most only a few simultaneous primary contacts can be established in proteins of length n ≥ n_op_, most of the tertiary structure contacts will still be defined by contacts at shorter range established in later folding phases [17]. Formation of the primary contact in the SCM defines the primary loop, which subsequently collapses through two-state kinetics [17]. The nucleation by an early primary contact has been referred to within the model as “Nature’s shortcut to protein folding” [17]. Short-range contacts established in later folding phases are defined by fluctuating shorter loops, called minimal loops in the model, which are expected to be of length n_min_ ≈ 15 amino acids [36]. Thus, within the model, the observed persistence length of loops in a native protein is expected to be significantly lower than n_op_ and closer to n_min_, in agreement with experimental observations [37]. It is important to bear in mind, however, that the SCM is concerned with the optimal sizes of loops in the fluctuating unfolded chain rather than with the topology of the fully folded protein. The final length of the topological elements of the 3D structure can vary from their open chain seeding loops, as contacts in the folded structure are refined by optimal packing, secondary structure formation, and establishment of all the relevant interactions [38]. Because proteins longer than ≈100 amino acids do not generally undergo complete two-state collapse [17] but rather fold through multi-step pathways, consistent simple physical reasoning implies that there is a limit to the size of the primary loop (i.e., ≈100 amino acids) that can successfully lead to the native SCM folding pathway. The physical basis of early non-local contact formation is shown in Figure 2.

The concept of folding nucleated by non-local contacts is not exclusive of the SCM, having arisen earlier in the context of the diffusion–collision model [39], the loop hypothesis [40], and in the energy landscape picture [41]. It furthermore has appeared in simulations of the transition state of two-state folding proteins [42]. Protein topology has been considered an essential element of folding mechanisms in a number of theoretical efforts [37,43,44,45,46]. The particular feature in the SCM is that the early non-local contacts are highly specific as in the loop hypothesis [40], and the SCM provides the means to determine their location from primary sequence information [17].

### 2.2. FITR for the Ebola Virus Matrix Protein VP40 N-Terminal Domain

Ebola virus (EBOV) infection is one of the deadliest viral diseases for humans, with fatality rates that can reach up to 90% [47]. EBOV is an encapsulated single-stranded negative RNA virus that belongs to the *Filoviridae* family [48]. EBOV infection is triggered by contact with the virus through the endothelial or epithelial surfaces, including those in the nose, mouth, eyes, and gastrointestinal tract [49]. EBOV is particularly lethal because it attacks several vital organs, including the spleen, liver, kidneys, and lungs, shutting down their functions [49]. Two vaccines for EBOV disease have been approved recently, Merck’s rVSV-ZEBOV, and Janssen’s Ad26-ZEBOV/MVA-BN-Filo [50]. Moreover, as of 2021, there were two approved treatments based on monoclonal antibodies, Ridgeback’s Ebanga and Regeneron’s Inmazeb [51]. Concern has been expressed as to the appropriate cost-effective strategy to deploy vaccination for a disease that is highly lethal yet of limited spreading potential [52].

The genome of the Ebola virus encodes seven structural proteins, namely, NP, VP35, VP40, GP, VP30, VP24, and L [53]. VP40 is the most abundantly expressed EBOV protein, making it an attractive target for drug design [32]. VP40 is involved in (a) defining and maintaining the overall shape of the viral particle by formation of the filamentous matrix for the virus [54] and (b) has also been shown to play a role in the budding of EBOV from infected cells [55]. It is reasonable to expect in the absence of the viral assembly process that the spreading of the virus to further host cells would be seriously impaired.

Protein VP40 is 326 amino acids long (1ES6) and is formed by two domains, the N-terminal domain (NTD: residues 1–195) and the C-terminal domain (CTD: 196–326). The two domains can adopt different arrangements with respect to each other [56], allowing VP40 to exist in different conformational states including hexameric and octameric states, modulating its function along the virus cycle [54,57,58,59].

The calculated possible primary contacts for the N-terminal domain of VP40 are listed in Table 1.

**Figure 3:**
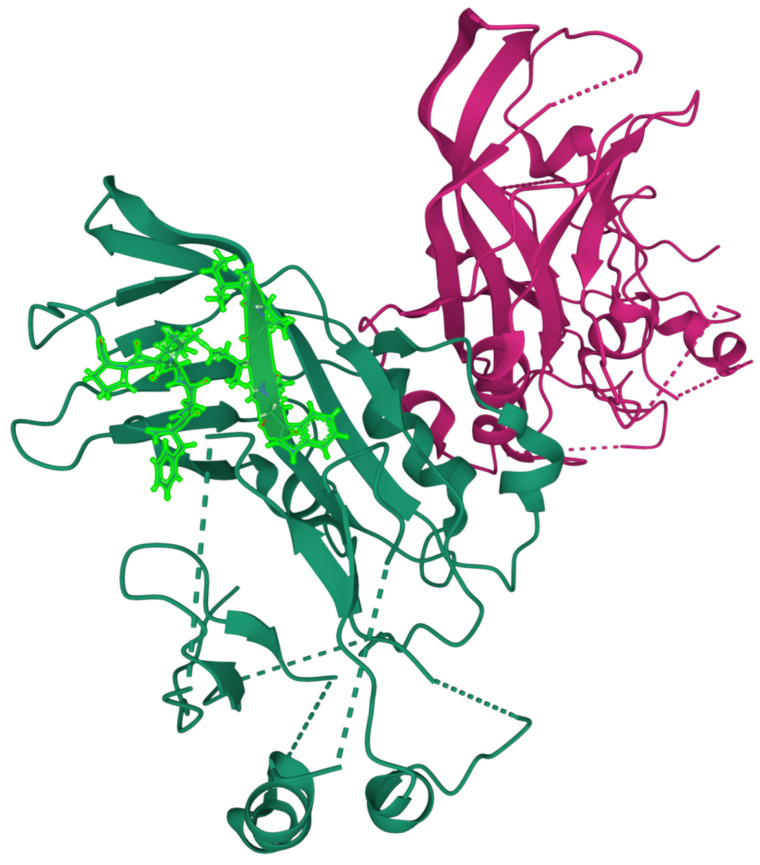
The best predicted primary contact in the native structure of VP40 (PDB structure 7JZT); the side chains of the amino acids included in the contact-defining segments are represented [62]. The structure is a dimer, and the primary contact has been represented only on a single chain for clarity.

The best primary contact is established between segments (^92^IPIWL^96^) and (^161^FVLPP^165^) with a predicted stability of ΔG_cont_ ≈ −14.8 *k*T. The best predicted primary contact is a good contact on the crystal structure [61] with side chains within Van der Waals interaction range, as shown in Figure 3. The location of the primary contact in the 3D structure is included only as an additional consistency test of the physical reality of the predicted contacts. As explained previously, the FITR strategy aims to interdict in the folding process in the unfolded state.

We also included the possible primary contacts whose stabilization energy is within ≈6 *k*T (i.e., whose populations are within ≈3 orders of magnitude of the best possible primary contact). Only the most stable contact is a good contact in the 3D structure, and thus it is natural within the SCM that it represents the entry point to “Nature’s shortcut to protein folding” [17], and most molecules are expected to fold through the pathway initiated by its formation. The calculations were performed following the methodology employed in the SCM previously [17] entailing a search for the most stable possible hydrophobic contacts between pairs of 5 amino acid segments i and j, located at a distance n_ij_ along the sequence such that 65 ≤ n_ij_ ≤ 100 amino acids (see the Section 3 for a complete explanation). 

On the basis of the above results, any of the two segments (^92^IPIWL^96^) or (^161^FVLPP^165^) could constitute a FITR for the N-terminal domain of VP40. Depending on which segment is chosen as the FITR, the other segment becomes a natural template to start the search for an adequate folding interdicting peptide (FIP). Thus, the best possible FIPs are (^161^FVLPP^165^) or (^92^IPIWL^96^). Moreover, both segments include highly conserved residues, suggesting that they might constitute mutation-resistant drug targets [63].

An interesting question is whether misfolding of just one of the two domains of VP40 would suffice in inactivating the protein’s functionality. Experiments and simulations show that rearrangement of the two domains into distinct conformational states is critical for VP40 to function [54]. A generally butterfly-like dimer configuration plays a role in the transport of the protein to the cellular membrane [64], while an hexameric form is the main element of the virus filament [64], and an octamer structure binds to RNA and regulates transcription [65]. Such rearrangements depend critically on interactions at the interfaces of the two domains and their flexibility [54]. Thus, it is reasonable to assume that broad configurational disruption of just one domain is likely to have serious deleterious consequences on viral functionality.

### 2.3. FITR for the Influenza a Virus M1 N-Terminal Domain

Influenza is one of the most common respiratory diseases affecting humans [19]. Many millions of infections occur every year, producing up to ≈65,000 deaths [21]. Moreover, influenza has pandemic potential and can induce potentially disastrous health crises such as the 1918 event that cost ~15 million lives [65]. The influenza virus is an enveloped, negative sense, single-stranded RNA virus with a segmented genome [66]. It has a broad avian zoonotic reservoir that ensures the continuous emergence of new virus variants [67]. Moreover, the influenza virus has high mutation rates and displays both antigenic shift and drift, making it very challenging to develop mutant-resistant treatments and vaccines [68]. Currently, vaccination remains the preferred strategy, with the vaccines periodically modified to overcome the virus seasonal variability, albeit with varying success [69]. Additionally, at least three therapeutics are currently used to treat influenza: oseltamivir, zanamivir, and peramivir [70], with other older treatments such as rimantadine and amantadine having been partially phased out due to the rise of antimicrobial resistance.

The matrix protein M1 of the filamentous strains of the influenza A virus is involved in defining and maintaining the overall morphology of the virus, thus being critical for its functionality, including virion assembly [71,72]. Reverse genetics experiments showed that when an influenza strain that is mostly spherical in shape (A/WSN/33 (H1N1)) is transfected with the M1 segment of a filamentous strain (A/Udorn/72 (H3N2)), the resultant recombinant virus acquires the ability to form the filamentous virus [73]. Moreover, the filamentous transfection-related transition requires both the N- and C-terminal domains of M1 [73], suggesting that disruption of the folding of a single domain should suffice to produce deleterious effects on the filamentous influenza strains [73].

M1 is a 252 amino acid protein that contains a N-terminal domain (residues 1–162) and a C-terminal domain (163–252) [74]. Because the C-terminal domain is only 89 residues long, we do not expect it to be necessarily amenable to SCM investigation, as the SCM contact predictions are focused on proteins longer than ≈100 amino acids with multi-state folding pathways rather than two-state folding kinetics [17]. 

Following the same procedure described above, we calculated the possible primary contacts for the N-terminal domain of the matrix protein M1 of the influenza A virus, and the results are listed in Table 2. 

**Figure 4:**
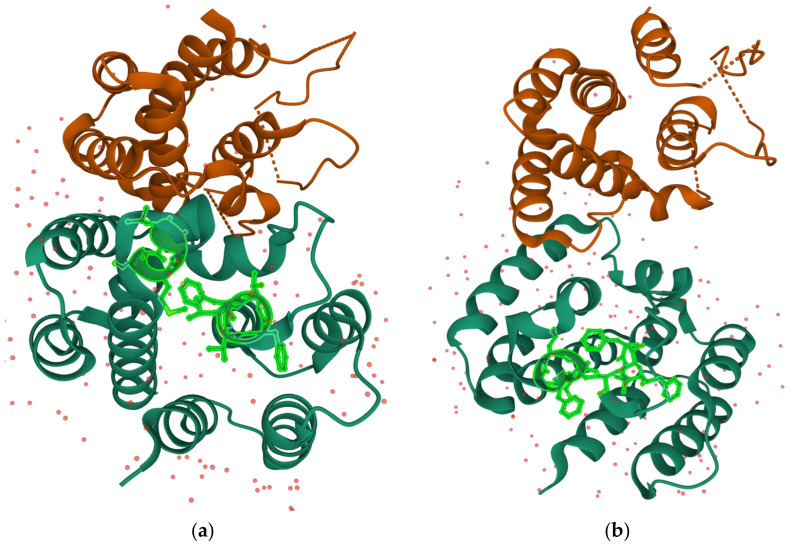
(**a**) The best predicted primary contact in the native structure of M1 [75]; (**b**) the second-best predicted contact is also represented. The side chains of the amino acids included in the contact-defining segments are represented. The structure is a dimer, and the primary contact has been represented only on a single chain for clarity. The pink dots are oxygen atoms of water molecules included in the crystal structure.

The best primary contact is established between segments (^62^FVFTL^66^) and (^127^CMGLI^131^) with a predicted stability of ΔG_cont_ ≈ −11.8 ± 0.2 *k*T. The best predicted primary contact is a good contact in the crystal structure [75] with side chains within Van Der Waals interaction range, as shown in Figure 4. 

Thus, we expect that this contact provides a good FITR for the N-terminal domain of M1. The second-best contact is established between segments (^62^FVFTL^66^) and (^144^FGLVC^148^), with a predicted stability of ΔG_cont_ ≈ −11.4 ± 0.2 *k*T, very close to the best one, and also involving segment (^62^FVFTL^66^). As shown in Figure 4 this is also a good contact in the 3D structure, and thus it constitutes the nucleation event for a second parallel folding pathway. 

The existence of two possible primary contacts in this case does not imply additional complexity when searching for an adequate FITR. Because both contacts share the segment (^62^FVFTL^66^), any folding interdicting peptide designed to specifically bind to this segment should disrupt the initiation of both folding pathways. The segments (^127^CMGLI^131^) and (^144^FGLVC^148^) constitute potential starting points from which to design an optimal FIP. Segment (^62^FVFTL^66^) is highly conserved [76], thus suggesting that any FIP drug that specifically targets it as a FITR would be useful against a broad range of influenza A mutants.

### 2.4. Additional Considerations on Designing Effective Therapeutic Drugs Based on the FITR

The potential advantages of the FITR strategy for the design of therapeutic drugs relies on at least three important considerations: (a) by aiming to interdict in the folding process early on, the FITR approach is less dependent than strategies based on targeting the native structure based on atomic level details that are hard to precisely determine [77]; (b) the segments involved in the primary contact provide natural templates from which to derive the therapeutic peptide, thus reducing the challenge of finding suitable therapeutic candidates [78]; and (c) because the protein segments involved in the nucleation of the initial folding events are likely to be highly conserved, the FITR is expected to show considerable resilience against viral escape through mutation [16].

Several challenges specific to FITR are also apparent when considering the practical application of the strategy: (1) designing a sufficiently specific peptide that effectively competes with intramolecular contact formation is a considerable challenge; (2) because the goal is to interdict the folding of a viral protein, the peptide has to be delivered to specific loci inside the cell (i.e., in the vicinity of the cell ribosomes); (3) a relevant question is also whether there should be a preference for any of the two segments involved in the primary contact as the template for the therapeutic drug according to physico-chemical (i.e., stability, helical content, etc.) or biological criteria. 

With respect to challenge 1, the segments predicted to constitute good FITRs will in general be quite hydrophobic, as they constitute the initiation location for folding. This might imply that any attempt to impair the viral protein folding process with an FIP based on a largely unmodified version of the segments included in the primary contact runs the risk of binding non-specifically to other molecules and structures in the cell, potentially inducing adverse side effects [79]. Possible approaches to overcome this potential difficulty could include (a) enlarging the FITR by including additional amino acids adjacent in the primary sequence to the original 5-residue segment, and (b) chemically modifying the FIP to make it more specific [78].

With respect to challenge 2, while peptides are in general membrane-impermeable, there is a growing number of techniques to facilitate the transfer of peptides across the cell membrane [80]. Relevant for the purposes of this paper, a number of cell-penetrating peptides (CPPs) have been identified [81]. 

With respect to challenge 3, it is important to recognize that the folding process must be interdicted in as early a stage as possible, as protein folding within the cell can be not only fast but can involve additional cellular machinery in the form of chaperones that could potentially interfere with the interdicting drug [82]. Thus, it is probably desirable to interdict the folding process by introducing a therapeutic drug that might attach to the relevant segment before ribosomal translation is complete [83]. This consideration would suggest that it is best to attempt the interdiction by employing the peptide closer to the C-terminus as the potential inhibitor, and the N-terminal one as the FITR. Delivering the therapeutic drug specifically close to the ribosome would probably require chemical modification [36], and it might be challenging to ensure that such modification does not interfere with the interdicting properties of the drug.

Finally, although the current application of the FITR strategy focuses naturally on peptide drugs, there is no limitation to developing non-peptide drugs that might have the same folding-interdicting effect. The effort to design of such non-peptide folding-interdicting drugs would probably benefit from the application of advanced molecular dynamics to optimize its binding to the FITR [84]. 

## 3. Methods: Determination of the Primary Contact in the SCM Model

The physical basis of the SCM and its most up-to-date formulation have been recently explained in full detail [17,85]. Here, only a brief summary of the methodology followed to determine the primary contact is presented.

On the basis of the model presented in the previous sections, whether there is a non-local contact in an otherwise unfolded state is dependent upon the stability of the potential contact candidates at loop length n_loop_, such that n_loop_ ≥ n_op_ amino acids. In the SCM, the stability of a contact formed by the number n_cont_ of amino acids, ΔG_contact_(n_cont_, n_loop_), can be written as

ΔG_contact_(n_cont_, n_loop_) ≈ ΔG_int,H_(n_cont_) + ΔG_loop_(n_loop_) + ΔG_cont,S_(n_cont_) (1)

Here, ΔG_loop_ represents the entropic free energy cost of the loop as discussed in Section 2.1. The term ΔG_int,H_ denotes all the enthalpic interactions that help stabilize the contact, possibly including hydrophobic interactions, Van der Waals interactions, hydrogen bonds, disulfide bonds, and salt bridges [38], and its value satisfies ΔG_int_ < 0. The term ΔG_cont,S_ > 0 represents the entropic cost of constraining the side chains of the amino acids defining the contact such that the contact is stable. A segment-specific determination of the value ΔG_cont,S_(n_cont_) for a given contact would require detailed MD techniques. However, a heuristic estimate can be made from earlier work within the SCM, which showed that the average entropic cost of folding per amino acid for a sample of 13 proteins was ΔG_folding/residue,S_ ≈ 0.85 *k*T/residue [86], and the maximum was ΔG_folding/residue,S_ ≈ 1.09 *k*T/residue. As these are estimates for the entropic cost for folding per residue of complete proteins that include highly buried as well as flexible exposed regions, it is then reasonable to expect that the entropic cost of a contact-forming region must be closer to the highest calculated values for ΔG_folding/residue,S_. Here, we assume that ΔG_cont,S_(n_cont_) for a contact including n_cont_ amino acids is approximately ΔG_folding/residue,S_, determined by the number of residues defining the contact, such that ΔG_cont,S_(n_cont_) ≈ 1.09 n_cont_. This result is clearly an approximation but suffices to establish a cut-off in the number of possible contacts that is consistent with the available structural data.

Hydrophobic interactions are well understood to constitute the main driving force of the folding process [38]. Other interactions such as hydrogen bonds are weaker [87], or as with disulfide bonds and salt bridges form later along the folding pathway [38]. Thus, for an early contact forming from the unfolded state, we can take ΔG_int_(n_op_) ≈ ΔG_hyd_(n_op_), where ΔG_hyd_(n_op_) represents the stabilizing effect of hydrophobicity in the early contacts, and Equation (1) can be written as

ΔG_contact_(n_cont_, n_loop_) ≈ ΔG_hyd_(n_cont_) + ΔG_loop_(n_loop_) + ΔG_contact,S_(n_cont_) (2)

Since the hydrophobic stabilization energy of the contact ΔG_hyd_ is determined by the hydrophobicity of the segments involved, the hydrophobicity values h_k_ are obtained from the Fauchere–Pliska scale [60] and assigned to each residue in accordance with previous calculations within the SCM. 

Because the amino acid side chains are significantly larger than the typical peptide bond length, early contacts between two hydrophobic amino acids will inherently involve segments, including several amino acids adjacent to the initial contact. The stability of this early hydrophobic contact will determine where the folding process is initiated. This picture is not unlike the zapping model of Dill and collaborators [88]. Here, the typical early contact segment size is taken to be ≈5 amino acids, in line with previous calculations within the SCM^14^. The 5 amino acid window size is based on the geometric considerations underlying the SCM [17]. In practice, within the SCM, the hydrophobicity h_k_ of each residue is added over a segment contact window of five amino acids centered at reside *i*, resulting in a segment hydrophobicity h_i_,_5_ (a value of ≈0.45 is equivalent to a change in energy of *k*T). The hydrophobicity value h_i,5_ is then added to the h_j,5_ value obtained for a 5 amino acid segment centered at residue *j*, located at a distance n_ij_ at least n_op_ amino acids apart along the sequence, and no longer than the maximum primary loop length of ≈100 amino acids, to give a contact stability of

ΔG_cont_(n_cont_, n_loop_) ≈ *k*T [−(h_i,5_ + h_j,5_)/0.45 + 3/2 ln n_ij_ + 10.9]  100 ≥ n_ij_ ≥ 65 (3)

## 4. Discussion

In this paper, we extended the previously proposed folding interdiction target region (FITR) strategy for the development of therapeutic antiviral drugs to the Ebola and influenza viruses. It was shown that the same methodology applied to determine potential targets for folding interdiction in SARS-CoV-2 can in principle be employed for other viruses. Specific peptide candidates were proposed from which to start the search for therapeutic drugs for both diseases. The broader applicability of the FITR strategy demonstrated here makes it reasonable to postulate that it might constitute a new avenue for the development of therapeutic drugs against a wide range of infectious diseases. There are considerable challenges to the practical development of effective in vivo drugs through the FITR strategy, and those would have to be overcome for any practical application. The final conclusion as to the usefulness of the FITR strategy depends on its experimental validation, and efforts in this regard are underway.

## Figures and Tables

**Table 1:** Predicted possible primary contacts for VP40. Error estimates are standard deviations [60]. The location on the structure of the defining segments was determined on the PDB structure 7JZT [61].

Contact	ΔG_cont_ (*k*T)	Contact on 3D Structure
^92^IPIWL^96^ on ^161^FVLPP^165^	−14.8 ± 0.2	Native
^92^IPIWL^96^ on ^168^LPQYF^172^	−12.0 ± 0.4	Non-native
^92^IPIWL^96^ on ^187^PAATW^191^	−11.4 ± 0.2	Non-native

**Table 2:** Predicted possible primary contacts for M1. Error estimates are standard deviations [60]. The location on the structure of the defining segments was determined on PDB structure 4PUS [75].

Contact	ΔG_cont_ (*k*T)	Contact on 3D Structure
^62^FVFTL^66^ on ^127^CMGLI^131^	−11.8 ± 0.2	Native
^62^FVFTL^66^ on ^144^FGLVC^148^	−11.4 ± 0.2	Native
^42^LMEWL^46^ on ^127^CMGLI^131^	−10.2 ± 0.3	Non-native
^51^ILSPL^55^ on ^127^CMGLI^131^	−9.6 ± 0.2	Non-native
^51^ILSPL^55^ on ^144^FGLVC^148^	−9.3 ± 0.2	Non-native
^42^LMEWL^46^ on ^115^IALSY^119^	−7.0 ± 0.3	Non-native

## Data Availability

Not applicable.

## Abbreviations

SCM: sequential collapse model.
